# Structural and Functional insights into the catalytic mechanism of the Type II NADH:quinone oxidoreductase family

**DOI:** 10.1038/srep42303

**Published:** 2017-02-09

**Authors:** Bruno C. Marreiros, Filipa V. Sena, Filipe M. Sousa, A. Sofia F. Oliveira, Cláudio M. Soares, Ana P. Batista, Manuela M. Pereira

**Affiliations:** 1 Instituto de Tecnologia Química e Biológica – António Xavier, Universidade Nova de Lisboa, Av. da Republica EAN, 2780-157 Oeiras, Portugal

## Abstract

Type II NADH:quinone oxidoreductases (NDH-2s) are membrane proteins involved in respiratory chains. These proteins contribute indirectly to the establishment of the transmembrane difference of electrochemical potential by catalyzing the reduction of quinone by oxidation of NAD(P)H. NDH-2s are widespread enzymes being present in the three domains of life. In this work, we explored the catalytic mechanism of NDH-2 by investigating the common elements of all NDH-2s, based on the rationale that conservation of such elements reflects their structural/functional importance. We observed conserved sequence motifs and structural elements among 1762 NDH-2s. We identified two proton pathways possibly involved in the protonation of the quinone. Our results led us to propose the first catalytic mechanism for NDH-2 family, in which a conserved glutamate residue, E_172_ (in NDH-2 from *Staphylococcus aureus*) plays a key role in proton transfer to the quinone pocket. This catalytic mechanism may also be extended to the other members of the two-Dinucleotide Binding Domains Flavoprotein (tDBDF) superfamily, such as sulfide:quinone oxidoreductases.

Type II NADH:quinone oxidoreductases (NDH-2s) are involved in respiratory chains of organisms belonging to the three domains of life, Eukarya, Bacteria and Archaea^1^. These are membrane associated enzymes which, by reducing quinones, indirectly contribute to the establishment and maintenance of the transmembrane difference of electrochemical potential. This potential is responsible for solute/nutrient cell import, synthesis of ATP and motility, *i.e.* it is vital for life.

NDH-2s are members of the two-Dinucleotide Binding Domains Flavoprotein (tDBDF) superfamily, a large group of proteins involved in several metabolic processes^2^. The tDBDF includes different families such as monooxygenases, glutathione reductases, dihydrolipoamide dehydrogenases, ferredoxin reductases and sulfide dehydrogenases. As its name implies, the members of this superfamily present two structural domains for the binding of dinucleotides. These domains are structurally similar to each other and each one adopts a Rossmann fold, known to stabilize the adenine rings of dinucleotides (Fig. 1A)^3^. The domain at the N-terminal binds the flavin prosthetic group, a flavin adenine dinucleotide (FAD), and the second domain interacts with either nicotinamide adenine dinucleotide (NADH) or nicotinamide adenine dinucleotide phosphate (NADPH). FAD is generally not covalently bound and its isoalloxazine ring is buried inside the protein, with its *re*-side facing the NADH binding domain (Fig. 1B). Sulfide:quinone oxidoreductase (SQR) and sulfide:flavocytochrome *c* oxidoreductase (also called flavocytochrome *c* sulfide dehydrogenase, FCSD) are exceptions within the superfamily because, although the two enzymes contain the two dinucleotide binding domains, they do not interact with NADH due to the presence of a loop, which makes the NADH binding site structurally inaccessible. Many members of the tDBDF superfamily have additional redox centres, in most known cases a disulfide, justifying the superfamily being also named flavin-disulfide reductases.

Structures of NDH-2 from the yeast *Saccharomyces cerevisiae*, also called Ndi1 (PDB:4G6H and PDB:4G9K)^4^,^5^, and those from bacteria, *Caldalkalibacillus thermarum* (PDB:4NWZ)^6^ and *Staphylococcus aureus* (PDB:4XDB^7^), have been determined. In addition to the two dinucleotide binding domains, all structures show the presence of a C-terminal domain with two amphipathic helices which allow NDH-2 to interact with the membrane (Fig. 1A). Moreover, the first domain was proposed to contain the motif AQxAxQ in the quinone binding site^6^. The structures are in general similar, but different results were obtained in the case of the structures of Ndi1 incubated with the substrates, NADH and quinone. The structure determined by Iwata *et al* suggests NADH and quinone would superimpose at the *re*-side of FAD^5^, while the structure solved by Feng and co-workers showed distinct binding sites for NADH and quinone at the *re*- and *si*-side of FAD, respectively^4^. The structures of the bacterial enzymes were obtained in the absence of the substrates, but biochemical and biophysical data provided evidences for the presence of different binding sites for the two substrates^6^,^7^. Sena *et al* detected, for NDH-2 from *S. aureus,* a charge-transfer complex formed between NAD^+^ and the reduced flavin, which is dissociated by the quinone^7^. Recently NDH-2 from *Escherichia coli* was also shown to have two distinct substrate binding sites and a bound semi-protonated quinol was identified as a catalytic intermediate^8^. All these recent findings were major advances for the understanding of NDH-2, but its overall mechanism is still unclear.

In this work we performed thorough sequence and structural analyses in which we identified relevant amino acid residues, sequence motifs and structural elements. The integrated data allowed us to identify common denominators of 1762 NDH-2 sequences and establish the basis to discuss and propose a universal catalytic mechanism for NDH-2.

## Results and Discussion

In this work we performed a thorough structural analysis of NDH-2s in order to identify structurally relevant elements and/or motifs, which helped to elucidate the poorly understood catalytic mechanism of these enzymes.

For analyses and discussion of the results, we used the amino acid sequence and tertiary structure of NDH-2 from *S. aureus* ([SA0802], PDB:4XDB^7^), unless otherwise mentioned.

### Amino acid residue conservation

We performed a multiple sequence alignment using 1762 NDH-2s and looked for highly conserved amino acid residues. In general, NDH-2s have on average 430 amino acid residues, 30 of which we observed to have conservation equal to or higher than 80%, *i.e.* these 30 amino acid residues are present at the same position in at least 80% of the analyzed sequences.

#### Conservation in the first dinucleotide binding domain: FAD binding site

**Figure 1:**
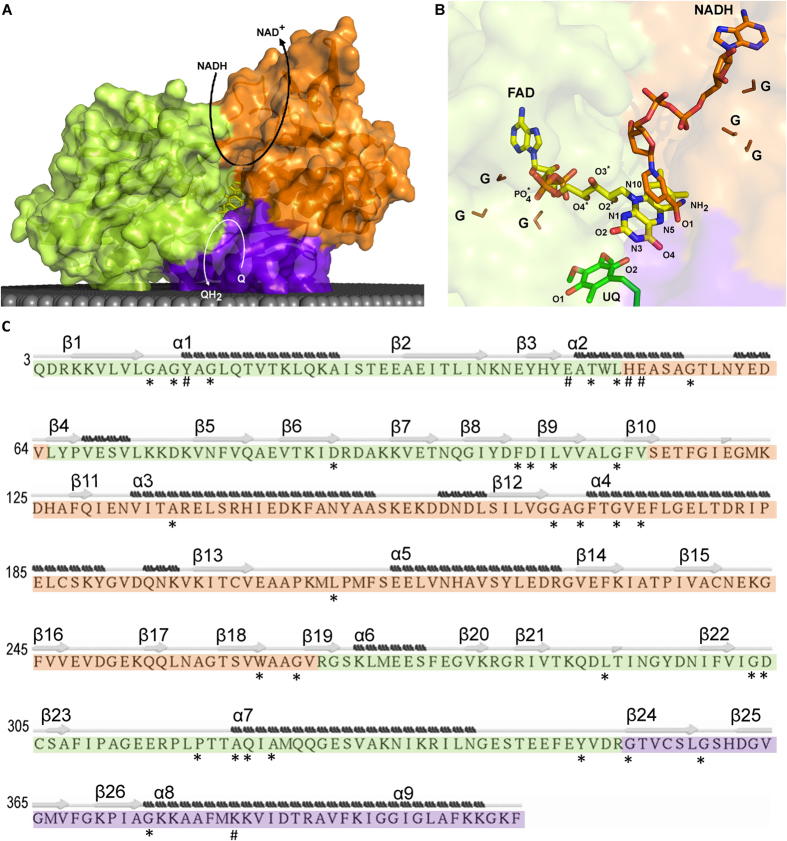
NDH-2 and substrates. NDH-2 is composed of three structural domains: first dinucleotide binding domain, or FAD binding domain (green); second dinucleotide binding domain or NADH binding domain (orange); and membrane interacting domain, including two amphipathic helices at the C-terminal (purple). (**A**) Cartoon representation of the X-ray crystal structure of NDH-2 from *S. aureus* (PDB:4XDB^7^). The gray area represents the membrane and curved arrows schematize NADH:quinone oxidoreductase activity; (**B**) Cartoon representation of a zoomed view of the FAD region and co-crystallized ubiquinone and NADH of the NDH-2 from *S. cerevisiae* (PDB:4G73^4^). The atoms of the FAD group are ordered and coloured in: blue – Nitrogen atom (N); red – Oxygen atom (O); orange – Phosphorus atom (P) and yellow – Carbon atom (**C**). The glycine residues composing the GxGxxG motif present each of dinucleotide binding domain are coloured in brown and indicated by “G”; (**C**) Sequence of NDH-2 from *S. aureus*^7^ indicating secondary structure elements. Secondary structure was predicted using STRIDE. β-sheets and α-helices are numbered from the N- to the C-terminal. Residues with at least 80% conservation (*) and with high covariance (#) are marked.

**Figure 2:**
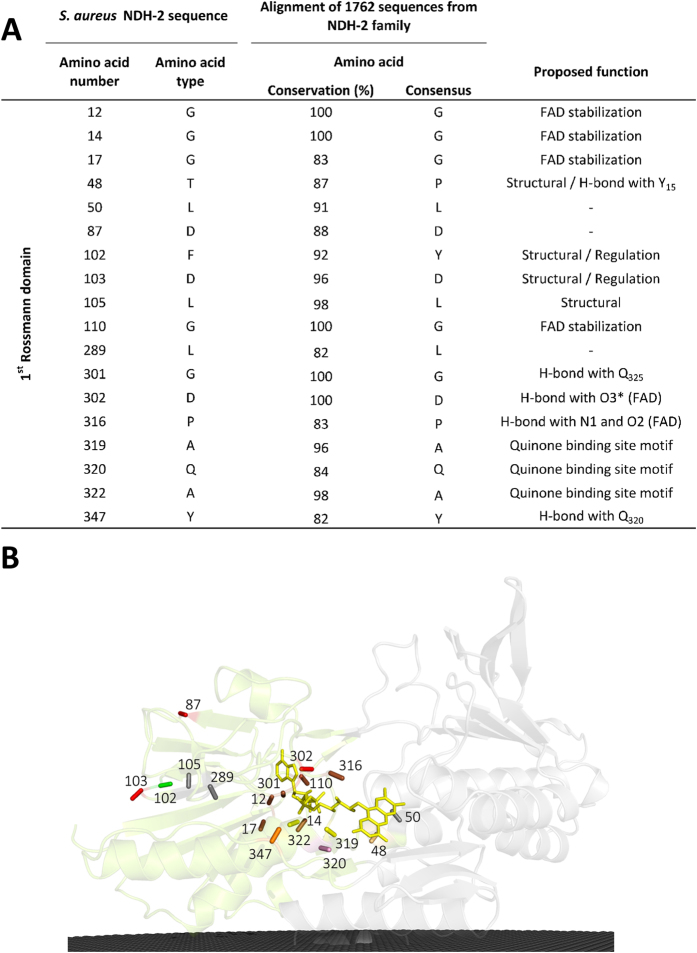
Amino acid residue conservation in the first dinucleotide binding domain. (**A**) List of the 18 amino acid residues present in the 1^st^ dinucleotide (FAD) binding domain that are conserved in at least 80% of the NDH-2s; (**B**) Cartoon representation of the X-ray crystal structure of NDH-2 from *S. aureus* (PDB:4XDB^7^) highlighting the location of the 18 conserved amino acid residues present in this domain. Membrane is represented in black.

Eighteen conserved amino acid residues are present in the first dinucleotide binding domain, arranged in four motifs and seven isolated residues (Figs 1 and 2). The first conserved motif observed is GxGxxG (G_12_xG_14_xxG_17_). In 9% of NDH-2s, the last glycine residue of this motif is substituted by an alanine residue, as in the case of the NDH-2 from *S. cerevisiae* (Ndi1)^4^,^5^. This glycine rich motif is placed in a loop located close to the pyrophosphate moiety of the FAD and should stabilize it (Figs 1B and 2)^3^,^6^,^9^.

The second conserved motif, located at the surface of the protein, is composed of the residue pair YD (F_102_D_103_ in *S. aureus*), the Y and D residues being present in 92% and 96% of the NDH-2s, respectively (Fig. 2). We observed that the tyrosine residue is replaced by a phenylalanine residue in 7% of the NDH-2s, as in the case of NDH-2 from *S. aureus* (F_102_). The conservation of the YD pair is intriguing because it is located far from the active centre and binding sites of the two substrates. As this pair is present between two β-strands (Fig. 1C) (β8 and β9), the hydrophilic character of the side chain of D_103_, that points towards the solvent, could constrain the position of β9 (part of the Rossmann fold) and therefore the pair might have a structural role in the Rossmann fold. However another Rossmann fold is present in the second dinucleotide binding domain without such a conserved pair. Thus alternatively and more appealing, the conserved YD pair may constitute a site for regulation of the enzymatic activity.

A third strictly conserved pair, G_301_D_302_, forming the third conserved motif, is observed close to O3* of FAD (Figs 1B and 2). The backbone of G_301_ establishes a hydrogen bond (2.7 Å) with the side chain of a residue located after the proposed quinone binding site motif (A_319_Q_320_xA_322_xQ_324_)^6^ in α-helix 7 (Fig. 1C). This residue is a glutamine in 57% of the NDH-2s, such as Q_325_ in *S. aureus*, and a glutamate and a histidine residue in the cases of NDH-2 from *S. cerevisiae* and *C. thermarum*, respectively^4^,^6^,^7^. D_302_ was previously suggested to make hydrogen bonds with FAD, both through its backbone to the PO_4_ group and its side chain to O3* (Fig. 1B)^5^,^6^,^10^,^11^. Studies performed with NDH-2 from *S. cerevisiae*, in which the equivalent aspartate residue was mutated (to alanine, asparagine, glutamine, or glutamate) showed that the presence of a glutamate/aspartate residue is important for the activity of the enzyme^12^. The high conservation of that aspartate residue (D_302_) is extended to several families of the tDBDF superfamily, even to those whose members do not interact with quinones (our unpublished results), suggesting that it could be important in the oxidation/reduction processes of the FAD.

We also observed that three out of the four first residues from the quinone binding site motif, AQxAxQ^1^,^6^, are more than 80% conserved (Fig. 2), and that the last glutamine residue is still present in 78% of NDH-2s. The backbone of A_319_ and Q_320_ was described before as making direct hydrogen bonds with the isoalloxazine ring of FAD^5^,^6^, while the two glutamine residues (Q_320_ and Q_324_) were proposed to be at the entrance to the active site. We investigated alternative quinone binding site motifs and observed three main motifs: AQxAxQ (already mentioned above), AQxAxR (wherein the last glutamine residue is replaced by arginine) and APxAxQ (wherein the first glutamine residue is replaced by proline). These three motifs are conserved in 62%, 15% and 10% of the 1762 NDH-2s, respectively^1^.

#### Conservation in the second dinucleotide binding domain: NADH binding site

**Figure 3:**
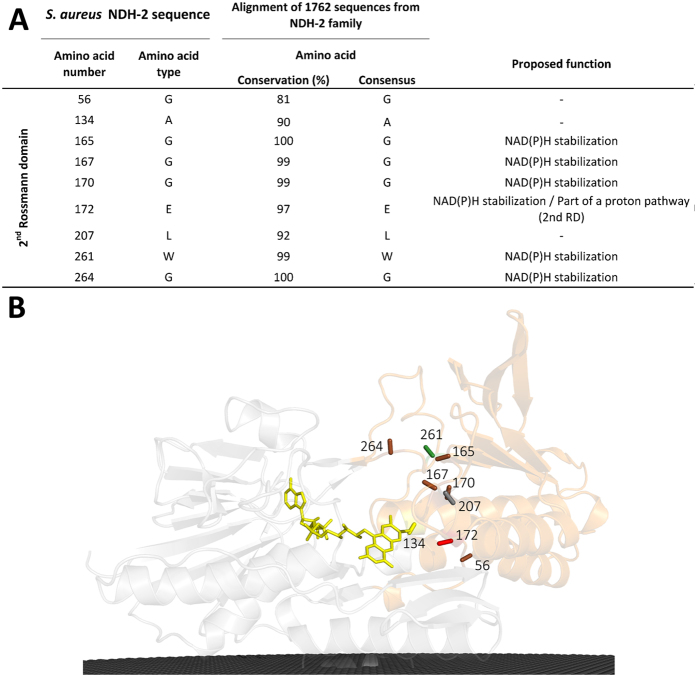
Amino acid residue conservation in the second dinucleotide binding domain. (**A**) List of the 9 amino acid residues present in the 2^nd^ dinucleotide binding domain that are conserved in at least 80% of the NDH-2s; (**B**) Cartoon representation of the X-ray crystal structure of NDH-2 from *S. aureus* (PDB:4XDB^7^) highlighting the location of the 9 conserved amino acid residues in this domain. Membrane is represented in black.

The second dinucleotide binding domain harbours the NADH binding site (Fig. 1A). In this domain, we identified nine conserved residues forming two different motifs plus three isolated residues (Fig. 3). The first conserved motif, GxGxxGxE, is located at the beginning of α-helix 4 (G_165_xG_167_xxG_170_xE_172_) (Fig. 1C). As in the case of the similar motif observed for the first dinucleotide binding domain, the glycine residues were proposed to stabilize the pyrophosphate moiety of the dinucleotide, now NADH^4^,^6^,^12^. Replacing the first glycine residue by serine hampered the growth of *S. cerevisiae*^12^. The glutamate residue E_172_ is at hydrogen bond distance from the co-crystallized NADH nicotinamide ring in the structure of NDH-2 from *S. cerevisiae*^4^ (Fig. 3). This residue is conserved in 97% of the 1762 NDH-2s, while a glutamine residue is present in the remaining sequences (3%, mainly Archaea). Single mutation experiments showed yeast cells had growth defects when that glutamate residue (E_242_ in NDH-2 from *S. cerevisiae*) was replaced by alanine or aspartate residues^4^. This mutation also affected NADH and quinone kinetic parameters (K_M_ and V_max_)^4^, suggesting an important role of this residue in the catalytic mechanism of NDH-2.

The motif WxxG (W_261_xxG_264_) is also highly conserved, 99% and 100% for W_261_ and G_264_, respectively (Fig. 3). As W_261_ is close to the adenine base of NADH we hypothesize that it is of importance in the orientation and/or stabilization of NAD(P)H.

#### Conservation in the C-terminal domain: Membrane interacting module

**Figure 4:**
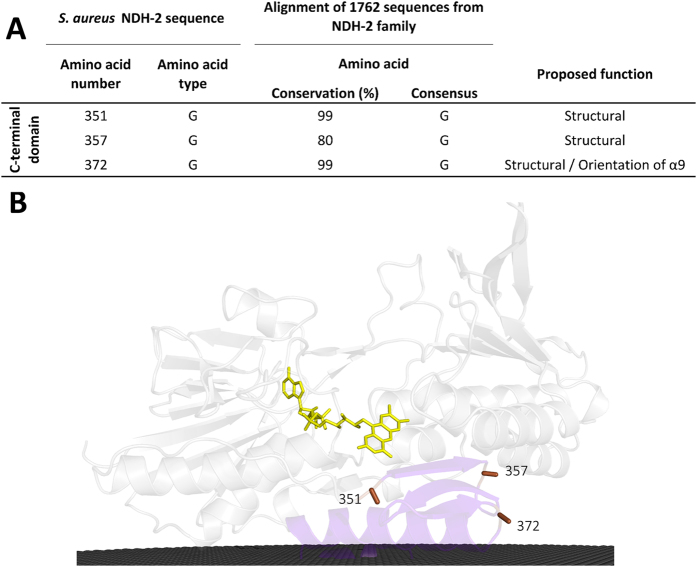
Amino acid residue conservation in the C-terminal domain. (**A**) List of the 3 amino acid residues present in the C-terminal domain that are conserved in at least 80% of the NDH-2s; (**B**) Cartoon representation of the X-ray crystal structure of NDH-2 from *S. aureus* (PDB:4XDB^7^) highlighting the location of the 3 conserved amino acid residues in this domain. Membrane is represented in black.

The C-terminal domain allows protein interaction with the membrane through two amphipathic α-helices (Fig. 1A and C)^4^,^5^,^6^,^7^. We found three conserved glycine residues (G_351_, G_357_ and G_372_),with G_372_ conserved in 99% of NDH-2s (Fig. 4) and we hypothesize that its presence is important to define the position of the first amphipathic α-helix in relation to the catalytic centre. By comparing the crystallographic structures of the members of the two families of quinone reducing proteins of the tDBDF superfamily (NDH-2^4^,^5^,^6^,^7^ and SQR^13^,^14^) we observed, in both cases, that the first amphipathic α-helix occupies the same position in relation to the isoalloxazine ring of the FAD. The localization of this α-helix allows the side chains of its amino acid residues to interact with FAD and substrates (NAD(P)H or sulfide and quinone).

### Amino acid residue covariance

Aiming to avoid excluding possibly relevant amino acid residues with lower conservation, we performed a covariance analysis using the MISTIC tool^15^. This tool gives insights into the relation between two residues by predicting positional correlations based on the structure and multiple sequence alignment. For example, during evolution, an amino acid residue at position “A”, important for the reaction, can be changed without loss of protein activity if a change in another amino acid residue at position “B” takes place, compensating for the change of the first amino acid at position “A”.

**Figure 5:**
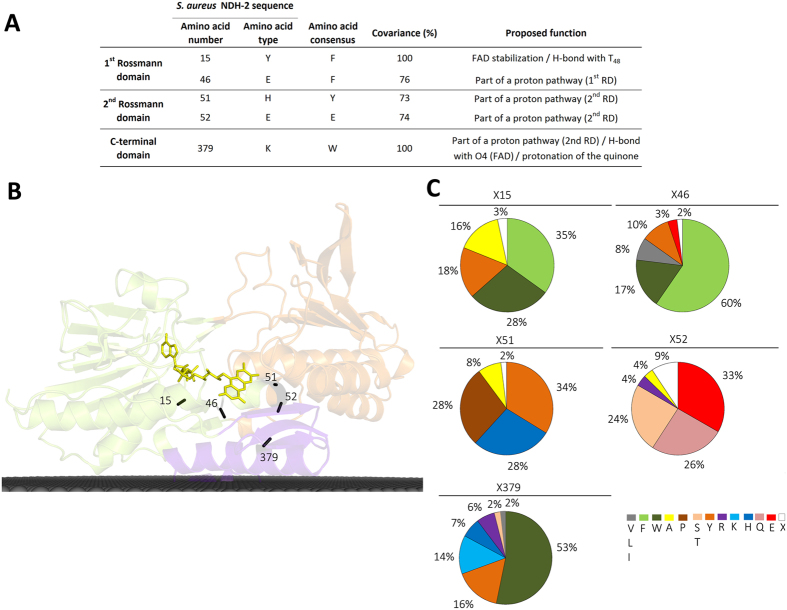
Amino acid residue covariance in NDH-2 family. (**A**) List of the 5 amino acid residue positions with the highest cumulative covariance (above 70%); (**B**) Cartoon representation of the X-ray crystal structure of NDH-2 from *S. aureus* (PDB:4XDB^7^) highlighting the location of the 5 amino acid residues positions with the highest cumulative covariance. Membrane is represented in black; (**C**) Amino acid residue frequency at the 5 amino acid positions with the highest covariance.

Our analysis revealed the existence of residues with high cumulative covariance, *i.e.* sum of all relations between a residue at a certain position and others at different positions. We selected all residues with cumulative covariance above 70%, when normalized in relation to those positions with the highest cumulative covariance, which were X_15_ and X_379_ (100% of cumulative covariance). Therefore, we accepted for analysis three additional positions: X_46_, X_51_, X_52_ (Fig. 5). Importantly, these five positions with the highest cumulative covariance establish covariance pairs between themselves, such as X_15_ with X_51_; X_52_ and X_379_; X_46_ with X_379_; X_51_ and X_52_ with X_15_ and X_379_. This observation further supports the structural/functional relevance of those amino acids, which are located in key positions, such as the NADH and quinone binding sites.

#### Covariance in the first dinucleotide binding domain: FAD binding site

The first dinucleotide binding domain contains two of the five positions with the highest cumulative covariance in NDH-2 family, X_15_ and X_46_ (Fig. 5). X_15_ (Y_15_ in NDH-2 from *S. aureus*) is part of the FAD binding motif, G_12_xG_14_Y_15_xG_17_. This position is occupied by an aromatic residue in 81% of NDH-2s, varying between a phenylalanine (35%), a tyrosine (18%, in NDH-2s from *S. aureus* and *C. thermarum*) or a tryptophan (28%, W_63_ in NDH-2 from *S. cerevisiae*) residue. In 16% of the cases, the conserved aromatic character is lost and replaced by an alanine residue (Fig. 5). X_15_ was previously described as being part of the tunnel extending from the C-terminal domain to the *si*-side of the FAD, and was able to establish a direct hydrogen bond, through its backbone, with one of the PO_4_ groups from FAD (Fig. 1B)^5^,^6^.

The second amino acid position with high cumulative covariance, X_46_ (E_46_ in *S. aureus*, see below), is also located at the *si*-side of FAD (Figs 1B and 5). This position is occupied by an aromatic residue (phenylalanine, tyrosine or tryptophan) in 87% of the NDH-2s (Fig. 5).

#### Covariance in the second dinucleotide binding domain: NADH binding site

The second dinucleotide binding domain contains two positions, corresponding to H_51_ and E_52_, localized at the *re-*side of FAD, among the five positions with the highest cumulative covariance (Figs 1B and 5). X_51_ varies mainly between three residues: tyrosine (34%), histidine (28%, in *S. aureus* and *C. thermarum*) or proline (28%, P_95_ in *S. cerevisiae*), while X_52_ (E_52_ in *S. aureus*) may contain a glutamate (33%), glutamine (26%, Q_50_ in *C. thermarum*) or serine (24%, S_96_ in *S. cerevisiae*) residues (Fig. 5 and Supplementary Figure 1). In the case of *C. thermarum*, we observed a glutamate residue also present in the vicinity (two residues before) of the histidine (H_49_) (corresponding to E_47_ in *C. thermarum*). These residue pairs (H_51_ and E_52_ in *S. aureus* and E_47_ and H_49_ in *C. thermarum*) seem to form a conserved motif that may have a role in the proton transfer process (the two residues composing the pair are at ~3.3 Å and ~3.9 Å apart, respectively). In NDH-2s from *S. aureus* and *C. thermarum*, H_51_ is also at hydrogen bond distance from the side chain of the highly conserved E_172_ (~3.3 Å), from the side chain of K_379_ (~3.3 Å) and near N5 from the FAD isoalloxazine ring (~6.8 Å in *S. aureus*) (Fig. 1B and Supplementary Figure 2). The analysis of protonation equilibrium simulations performed for NDH-2 from *S. aureus*, showed that H_51_ is sensitive to the oxidation state of FAD (Supplementary Table 1).

#### Covariance in the C-terminal domain: membrane interacting module

X_379_ (K_379_ in *S. aureus*), also included in the five positions with the highest cumulative covariance, is located in the C-terminal domain (Fig. 5). X_379_ is a tryptophan residue in 53% of NDH-2s (W_478_ in *S. cerevisiae*), a positively charged residue (K, H or R) in 27% (K in *S. aureus* and *C. thermarum*), or a hydroxyl containing residue (16% tyrosine and 2% threonine) (Fig. 5 and Supplementary Figure 1).

### Identification of two distinct proton pathways

The catalytic steps in NADH:quinone oxidoreduction, *i.e.* NADH oxidation, FAD reduction, FADH_2_ oxidation and quinone reduction involve proton transfers. Therefore, we looked for possible proton pathways, examining the conservation of amino acid residues by type (*e.g.* protonatable and aromatic,) and analyzing the three available NDH-2 structures^4^,^5^,^6^,^7^. We were able to identify two distinct proton pathways.

#### A proton pathway in the second dinucleotide binding domain: NADH binding site

**Figure 6:**
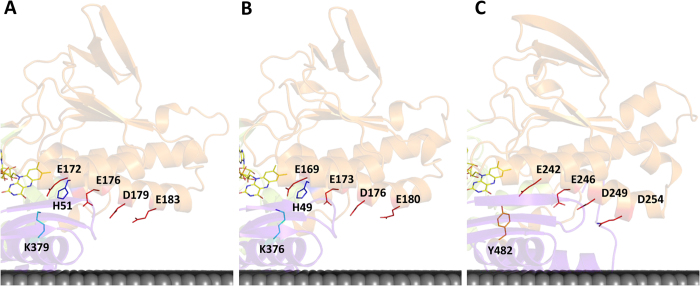
Proton pathways in the second dinucleotide binding domain. Cartoon representations of the X-ray crystal structures highlighting the proton pathways proposed for NDH-2s from (**A**) *S. aureus* (PDB:4XDB^7^); (**B**) *C. thermarum* (PDB:4NWZ^6^); (**C**) *S. cerevisiae* (PDB:4G73^4^). Membrane is represented in black.

On the *re*-side of FAD, we observed that the conserved E_172_ is at hydrogen bond distance from several residues and possibly from the -NH_2_ group of the nicotinamide ring of NAD(P)H. The side chain of E_172_ may establish three different hydrogen bonds with residues in its vicinity, namely with H_51_, and the backbone of S_355_ and K_379_ (Supplementary Figure 2), among which X_51_ and X_379_ are the positions with the highest cumulative covariances. In the three available NDH-2 structures, we noticed the glutamate residue is located at the interior end of a wire composed mainly of carboxylate residues connected to the surface of the protein (Fig. 6). All these carboxylate residues have their side chains oriented to the same side of α-helix 4 (Fig. 1C). These residues are E_172_, E_176_, D_179_ and E_183_ in NDH-2 from *S. aureus* (Fig. 6A, respective distances are shown in (Supplementary Table 2)), E_169_, E_173_, D_176_ and E_180_ in NDH-2 from *C. thermarum* (Fig. 6B) and E_242_, E_246_, D_249_ and D_254_ in NDH-2 from *S. cerevisiae* (Fig. 6C), and have an overall conservation of 97%, 62%, 74% and 28%, considering the conservation of the carboxylate residues *i.e.* glutamate or aspartate.

We performed protonation equilibrium simulations for NDH-2 from *S. aureus* (Supplementary Table 1), which clearly showed that the protonation of E_172_ (E_242_ in *S. cerevisiae*, Supplementary Table 3) is highly dependent on the oxidation state of FAD. E_172_ is the residue with the highest variation of its protonated fraction when comparing the reduced and oxidized states (Supplementary Table 1). These results support the idea that E_172_ is likely to play a role in proton transfer during the catalytic cycle.

The proton wire just described connects the surface of the protein and the NADH binding pocket. However, we hypothesize that this wire may be extended to the quinone binding pocket due to the presence of K_379_, with which E_172_ may interact (~3.3 Å) through a hydrogen bond (Fig. 5). X_379_ is located close to the isoalloxazine ring of FAD (at ~3.3 Å from its O4) and at the interface between the NADH and quinone pockets. However, we noticed 53% of NDH-2s do not contain a proton conductive residue at X_379_, but in 51% and 46% of these cases we observed a tyrosine or a histidine residue, respectively, at position X_383_ (corresponding to Y_482_ in *S. cerevisiae* at ~3.1 Å from E_242_) (Supplementary Figure 1), whose side chain seems to occupy the same structural position as that of K_379_ from *S. aureus* (structural alignment between NDH-2s from *S. aureus* and *S. cerevisiae* [RMSD = 1.2 Å]). Considering together the X_379_ and X_379+4_ (X_383_) positions in the NDH-2 alignment, we observed 98% NDH-2s have a proton conducting residue at the interface of the NADH and quinone pockets (X_379_/X_383_, Supplementary Figure 1), directly interacting with E_172_. Thus, in 98% of NDH-2s the proton wire present at the second dinucleotide binding domain may connect the protein surface and the quinone pocket.

We extended our analyses to SQRs, which are the only members of the tDBDF superfamily to have quinone as substrate, as in NDH-2s. SQR from *Aquifex aeolicus*^13^ presents hydrogen bonds between position X_172_ and X_51_ and X_379_ (Supplementary Figure 3). This reinforces the proposal for the presence of a proton conducting residue at the interface of NADH/sulfide and quinone pockets for these two families. As both families share the same electron acceptor, we may hypothesize that the residues occupying positions X_51_ and X_379_/X_383_ have a role in quinone protonation, possibly as proton conducting elements.

In summary, we propose the existence of a conserved proton conductive wire from the protein surface into the quinone pocket (Fig. 6), which certainly has an important role in proton transfer during the catalytic cycle. The wire is established by a sequence of conserved carboxylic residues E_172_/E_176_/D_179_/E_183_, intercalated by H_51_, to K_379_ or its structural equivalent (X_383_), (Fig. 5). Other possibilities for proton conductive wires are shown in Supplementary Figure 4.

#### A proton pathway in the first dinucleotide binding domain: FAD binding site

**Figure 7:**
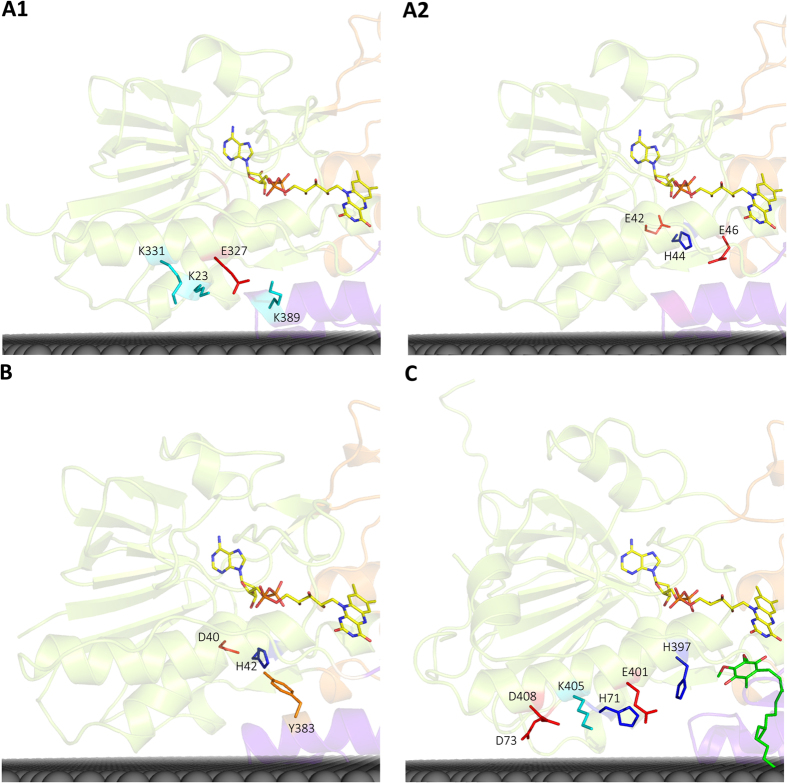
Proton pathways in the first dinucleotide binding domain. Cartoon representations of the X-ray crystal structures of NDH-2 highlighting the (**A1**) proton pathway through α-helices 1 and 7 of NDH-2 from *S. aureus* (PDB:4XDB^7^); (**A2**) alternative proton pathway in NDH-2 from *S. aureus* (PDB:4XDB^7^); (**B**) the proton pathway found for NDH-2 from *C. thermarum* (PDB:4NWZ^6^); (**C**) Proton pathway through α-helices 1 and 7 of NDH-2 from *S. cerevisiae* (PDB:4G73^4^). Membrane is represented in black.

In contrast to what was observed for the second dinucleotide binding domain, there is no clear proton conductive wire composed of highly conserved amino acid residues in the first dinucleotide binding domain. Therefore, we searched for protonatable residues close to the quinone pocket. In the case of NDH-2 from *S. cerevisiae*, a histidine residue at the binding site motif, AQxAH_397_Q, is observed at 5.4 Å from the quinone^4^,^5^. Site directed mutations of this histidine residue led to hampered growth of yeast cells, suggesting its importance in protein function^4^. These observations led us to hypothesize H_397_ could be a direct proton donor to the quinone. Consequently, we identified a putative proton wire involving E_401_ at 3.5 Å from H_397_, H_71_ at 4.4 Å from E_401_, and another three residues that could interact with H_71_ upon rearrangement of the respective side chains (D_73_, K_405_ and D_408_) (Fig. 7C). However that histidine residue is not present in NDH-2s from *S. aureus* (AQxAM_323_Q) or from *C. thermarum* (AQxAI_320_Q), and is only present in 17% of NDH-2s, mainly in proteobacteria and some eukaryotic species.

Based on the hypothesis that the quinone binding pocket is located in the same place in all NDH-2s, we searched for residues whose side-chains spatially occupy the position of that of H_397_ in NDH-2 from *S. cerevisiae* and we identified three positions (Supplementary Figure 5). In NDH-2 from *S. aureus* we identified K_389_ as a candidate to replace H_397_ (*S. cerevisiae*) and we noticed the presence of a possible wire involving E_327_, K_23_ and K_331_ (Fig. 7A1). Three other residues may also form a proton wire to the quinone binding pocket, namely E_42_, H_44_ (present in 53% of the NDH-2s) and E_46_ (present in 3% of the NDH-2s, Fig. 7A2). In fact, this alternative is also observed in the protein from *C. thermarum* (Fig. 7B). We observed a histidine (H_42_, *C. thermarum*) in the place of H_44_, as well as a tyrosine (Y_383_, *C. thermarum*) which makes a hydrogen bond with H_42_ (2.9 Å), suggesting that the tyrosine may play the same role as E_46_ from *S. aureus* (Fig. 7A2 and B). Moreover, we note the presence of a glutamate or an aspartate residue (E_42_/D_40_) two positions before the histidine (H_44_/H_42_, *S. aureus* and *C. thermarum* respectively). This alternative proton pathway seems to be absent in *S. cerevisiae* since no histidine is present and a tryptophan present in the GxGxW_64_G motif seems to block that path to the quinone binding pocket.

Overall the proton wire present in the first dinucleotide binding domain is less evident and alternative paths could be considered, some of which are indicated in Supplementary Figure 5. Since the quinone substrate may have different chemical structures, we may speculate that the different conductive proton pathways may reflect different structural arrangements related to the nature of quinones used.

### Hypothesis for the catalytic mechanism

**Figure 8:**
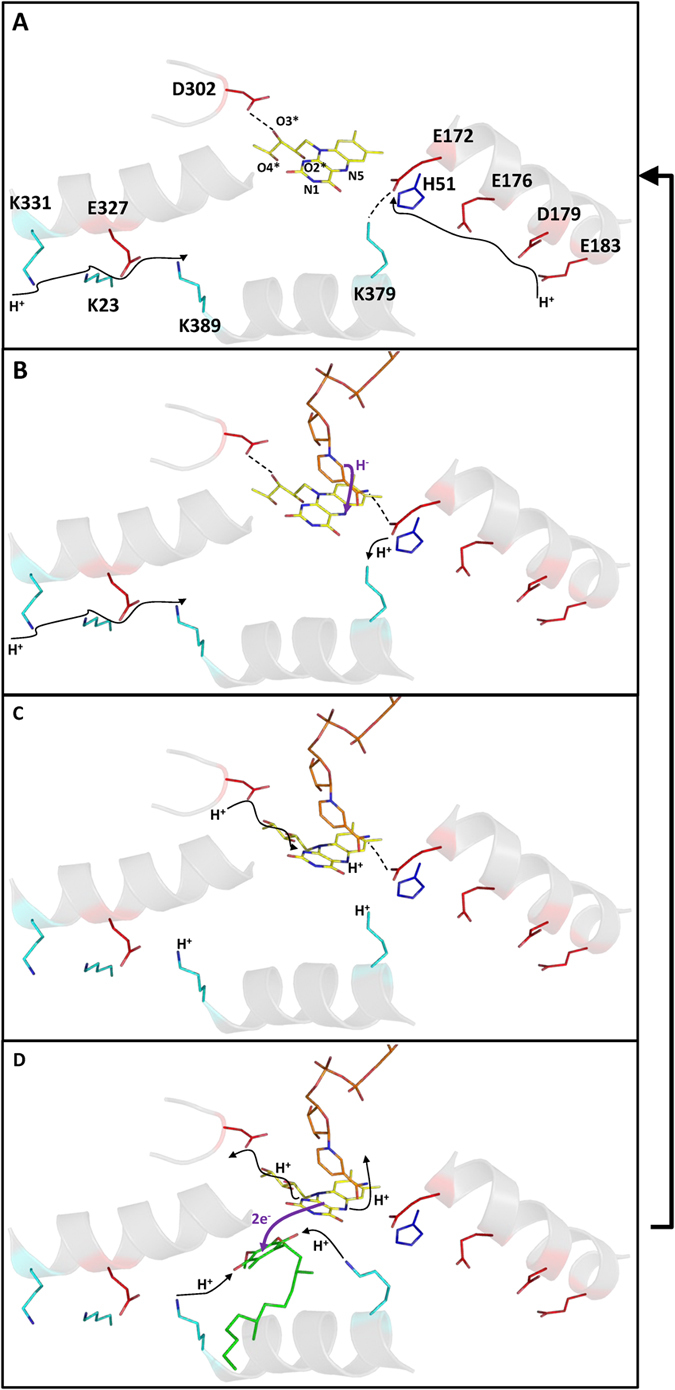
NDH-2 enzymatic mechanism. Illustrative representation of NDH-2′s enzymatic mechanism. Cartoons are based on the X-ray crystal structure of NDH-2 from *S. aureus* (PDB:4XDB^7^) and are a zoomed view of the FAD and substrate binding sites, including the helices (in gray) which contain the amino acid residues suggested to compose the proposed proton pathways. Substrate positions were predicted by superimposing the substrate free *S. aureus* and NADH/quinone bound *S. cerevisiae* NDH-2s structures (RMSD 1.2 Å). Sticks represent: yellow, the FAD; orange, the NADH/NAD^+^; green the quinone/quinol; red, glutamate/aspartate residues; cyan, lysine residues and dark blue, histidine residues. Hydrogen bonding interactions are represented by dashed black lines, proton transfers are schematized by the filled black arrows and electron/hydride transfers are indicated by purple filled arrows. (**A**) In the absence of substrates FAD is kept oxidized. In this case, D_302_ interacts with O3*. 1^st^ and 2^nd^ dinucleotide binding domain proton pathways allow proton conduction between the bulk and K_389_ or E_172_, respectively. E_172_ is at hydrogen bonding distance from K_379_; (**B**) Upon binding, NADH reduces FAD by hydride transfer to N5 and establishes a hydrogen bond with E_172_, which consequently loses the hydrogen bound to K_379_ (now protonated); (**C**) Concomitantly with its reduction, FAD adopts a bent conformation, leading to the rotation of O2*, O3* and O4*, changing the hydrogen bonding network between D_302_ and N1, allowing their interaction and protonation of N1 by D_302_. This conformation may also induce additional changes at K_389_ to adopt a protonated form close to the quinone binding pocket; D) Upon quinone binding, FADH_2_ transfers two electrons to the quinone which also accepts two protons from the final proton conductors of the pathways (K_379_ and K_389_). After the two electrons transfer (FADH_2_ oxidation), the flavin returns to its original conformation, leading to the release of the proton at N5 (NADH binding pocket) and the proton at N1 in a reverse process that restores the initial hydrogen bonding network around D_302_. NAD^+^ and quinol are released and the initial positions of K_379_ and K_389_ restored. The protein returns to the state described in (**A**).

The catalytic mechanism of NDH-2 is still unclear, even considering the available structural and functional data^4^,^5^,^6^,^7^,^8^,^12^. Nevertheless the gathered information showed that the two substrates bind to different sites, and that a charge-transfer complex is formed between NAD^+^ and the reduced flavin (FADH_2_), which is dissociated by the quinone^7^,^8^,^12^. Here, we discuss the possible roles of the conserved elements in the catalytic process (Fig. 8A), including in proton transfer and substrate interaction. We divide the discussion in two parts corresponding to the two half-reactions: FAD reduction (by NADH) and FADH_2_ oxidation (by quinone).

#### FAD reduction: first half-reaction

The way in which FAD is reduced in NDH-2 is unknown, but, based on what is observed for several flavoproteins, we consider that FAD is reduced by hydride transfer from NADH at its *re*-side^2^. Therefore, N5 of the FAD isoalloxazine can accept the hydride from C4 of the nicotinamide ring of NADH (which is at ~3.4 Å in the structure of NDH-2 from *S. cerevisiae*) (Fig. 8B).

The origin of the second proton needed for the full protonation of FAD is uncertain, nevertheless it can be assumed to occur at the N1 atom of FAD (Fig. 1B). Inspecting the vicinity of N1 we noticed the presence of the conserved D_302_, although not at proton binding distance to it (~7–8 Å, Fig. 1B). The fact that D_302_ is totally conserved, even among other members of the tDBDF superfamily, and present in the vicinity of FAD suggests its involvement in the second protonation of the flavin. This hypothesis is corroborated by the protonation equilibrium simulations performed for the *S. cerevisiae* enzyme which showed that the protonation of D_383_ (equivalent to D_302_ in *S. aureus*) is greatly influenced by the presence/absence of NAD^+^ at the catalytic site (Supplementary Table 3). The protonated fraction of D_383_ increases 14% at pH 7 when the complex FADH_2_-NAD^+^ is formed, as compared with the oxidized FAD.

Considering that the members of the tDBDF superfamily are structurally similar, they are likely to share the same protonation mechanism. In the cases of NADH:ferredoxin oxidoreductase and thioredoxin reductase, the isoalloxazine ring of the reduced FAD adopts a bent conformation (the so-called boat conformation) upon reduction, which contrasts with the planar conformation observed in the oxidized form^10^,^11^. The bent conformation causes the rotation of C2* which indirectly allows O2* to reorient in between D_302_ and N1 from FAD (Fig. 1B), establishing a new hydrogen bond network (Fig. 8C). This proton network may lead to protonation of N1 by D_302_.

In summary, NADH binds to NDH-2 and reduces FAD through hydride transfer to N5. The fully protonated state of the flavin is achieved by rearrangement of the hydrogen bond network around N1 induced by the adoption of a bent conformation by the isoalloxazine ring. We propose that this proton network rearrangement may involve the strictly conserved D_302_, which has direct access to the bulk (Fig. 8A,B and C).

#### FADH_2_ oxidation: second half-reaction

The second half-reaction involves electron transfer from FADH_2_ to the quinone, deprotonation of FADH_2_ and quinone protonation. Two possibilities for the whole process may be considered: (1) The quinone can be reduced directly by hydride transfer from FADH_2_, in this way needing only a second proton; (2) or the quinone reduction and protonation events occur separately.

The first hypothesis cannot be discarded in the light of the current experimental knowledge, but considering that what is conserved is important to the function of an enzyme family, including the presence of two proton conductive channels leading to the quinone pocket, we propose the second half-reaction of NDH-2 is best described by the hypothesis involving the transfer of two protons to the quinone.

FADH_2_ is oxidized by the quinone (interacting at the *si*-side) and the two protons are also released from the flavin. The deprotonation of N1 proceeds by rearrangement of the hydrogen bond network due to a conformational change of FAD from the bent back to the planar conformation upon reoxidation. The loss of the bent conformation and consequently of the hydrogen bond network involving D_302_, O2* and N1 of FAD, results in deprotonation of N1, in a reverse process to that described for the protonation of FAD (Fig. 8D). The release of the second proton may occur concomitantly with the release of NAD^+^ which leaves FAD directly connected to the bulk (Fig. 8D).

Simultaneously with the quinone reduction, the protonation of both its oxygen atoms (O1_q_ and O2_q_) occurs, involving the two proposed proton conducting pathways (Fig. 8D). O1_q_ is oriented to the proton conductive pathway present in the first dinucleotide binding domain (Fig. 1B), hence its protonation is likely performed by this pathway. This previously identified proton wire is able to conduct protons from the bulk to H_397_, K_389_ and Y_401_ for *S. cerevisiae, S. aureus* and *C. thermarum* respectively (at 5.4 Å in the case of H_397_), which will be the direct proton donors of O1_q_ (Figs 7 and 8D). O2_q_ is oriented to the proton conductive pathway at the second dinucleotide binding domain which is responsible for taking up protons from the bulk to E_172_ and then to position X_379_/X_383_ (Fig. 6), which is occupied by the final proton donors for O2_q_. In the oxidized state (Fig. 8A) X_383_ (H_397_ in *S. cerevisiae*) is at 6–7 Å from O2_q_, a distance that does not allow a direct proton transfer and thus conformational rearrangements have to be considered. We propose that, concomitantly with the NADH binding and FAD reduction, H_397_ (in *S. cerevisiae*) suffers an adjustment of its side chain. As described above for the first half-reaction, upon reduction, FAD adopts a bent conformation that may induce structural changes in α-helix 7 (which includes the quinone binding motif with H_397_ in *S. cerevisiae*) (Supplementary Figure 6). This idea strongly suggests that FAD reduction may be a requirement for the protein to adopt the necessary conformational state for quinone protonation by the first dinucleotide binding domain proton pathway. In fact, a similar situation may also occur in the second dinucleotide binding domain, where the side chain of E_172_ undergoes a conformational change upon formation of the FADH_2_-NAD^+^ complex, allowing its hydrogen interaction with X_379_/X_383_ to be disrupted, leading to protonation of O2_q_ by the protonated X_379_/X_383_ (Fig. 8D).

In summary, we propose that the reactive quinone oxygens O1_q_ and O2_q_ are protonated by the two proton pathways identified and described in this study. The proton at N5 atom from FAD is released to the bulk (through the NADH binding pocket) while that from N1 returns to D_302_ through a reverse process to that described for the protonation of FAD.

## Conclusion

We performed an exhaustive bioinformatic analysis in order to identify the relevant amino acid residues and structural elements within the NDH-2 family. We carried out this analysis in NDH-2s with recognized quinone binding motifs, *i.e.*_~_70% of the 2567 proteins considered members of the NDH-2 family^1^. We identified 30 amino acid residues conserved in at least 80% of the NDH-2 sequences (Figs 2, 3 and 4) and we recognized five positions with high cumulative covariance (X_15_, X_46_, X_51_, X_52_ and X_379_) (Fig. 5). Combining the conservation/covariance analyses and the information of the available structures from three NDH-2s^4^,^5^,^6^,^7^, we were able to identify relevant elements, such as one proton pathway in each dinucleotide binding domain. The proton pathway from the second dinucleotide binding domain (NADH binding) is more conserved among the NDH-2 family (Fig. 6) than that observed in the first dinucleotide binding domain (Fig. 7) and is composed of several glutamate or aspartate residues always leading to a proton conductive residue at X_379_/X_383_. Both pathways conduct protons from the surface of the protein to the quinone pocket. The localization of the two proton pathways suggests the quinone pocket may receive protons at both sides of its reactive oxygens. Moreover, the highly conserved E_172_ (present in 97% of NDH-2 sequences) seems to be part of the proton pathway present at the second dinucleotide binding domain (NADH binding) and may have a role in the coordination of the proton transfer. We suggest that E_172_, by interacting with the NH_2_ group from the nicotinamide ring of NADH, may alter hydrogen bonds with amino acid residues present in the vicinity, namely at positions X_51_ and X_379_/X_383_. The change in hydrogen bonds may trigger other conformational changes allowing proton transfer from X_379_/X_383_ to the quinone with consequent protonation (Fig. 8D).

As observed for other members from the tDBDF superfamily, we suggest that FADH_2_ undergoes conformational changes upon reduction by NADH that affect conserved residues at the first dinucleotide binding domain (FAD binding), namely the conserved GD and the quinone binding site motifs (which includes H_397_ in *S. cerevisiae*). The rearrangement of side chain residues for the stabilization of FADH_2_ may induce changes in β3 and α-helices 1 and 7 and trigger quinone protonation (Fig. 8D).

Curiously, amino acid sequence insertions, including EF-hand or CxxC motifs^1^, are observed in several NDH-2s between the conserved residues that form the GD motif and the next α-helix (α7)^1^. The EF-hand motif, for example, was proposed to regulate the NDH-2 activity in a calcium dependent manner^16^. These motifs may constitute sites for regulation of enzyme activity by acting on the residues that stabilize/protonate FAD in different oxidation states. Also, the distribution of NDH-2s based on key residues such as X_51_, X_379_ and X_383_ may be related with the type of quinone present in the catalytic reaction of NDH-2 and can give insights into the metabolic pathways in which NDH-2 is involved, since several species have more than one type of NDH-2 (Supplementary Figure 1).

The functional mechanism of NDH-2 here proposed constitutes a solid model to foster debate and inspire the design of future experimental approaches aimed at understanding the catalytic mechanism of NDH-2 as well as that of other members of the tDBDF superfamily.

## Material and Methods

### Sequence analysis

We have previously used the KEGG database to identify and select the members of the NDH-2 family (2567 NDH-2s). We performed the respective taxonomic analysis and observed that NDH-2 family is distributed in four main branches which we called groups A to D^1^.

In this work we opted to analyse the enzymes with the typical quinone binding site (AQxAxQ), or its alternatives (AQxAxR and APxAxQ). We aligned the remaining 1779 NDH-2 sequences (~70% amino acid sequences from the NDH-2 family) using PROMALS3D^17^. We manually refined our data set using Jalview 2.8.1^18^ for which we took into account three criteria: (a) existence of two GxGxxG like motifs for interaction with FAD and NAD(P)H and included few variations, namely the GxGxxA motif; (b) presence of a C-terminal amino acid extension for membrane interaction (C-terminal domain), and (c) absence of possible other domains fused at the N- or C-terminal. Our final data set included, in this way, 1762 amino acid sequences (distributed in the three domains of life, Eukarya, Bacteria and Archaea). Covariance between amino acid residues in NDH-2 family was determined using MISTIC^15^.

### Secondary and tertiary structure analyses

The crystallographic structures used as templates were those from *S. aureus* (PDB:4XDB^7^), *C. thermarum* (PDB:4NWZ^6^) and *S. cerevisiae* (PDB:4G73^4^). Images of the structures were generated using PyMOL Molecular Graphics System, Version 1.4, Schrödinger, LLC. Secondary structure of NDH-2 from *S. aureus* was predicted using Stride^19^. All distance measurements presented below were performed between the closest hydrogen atoms of both objects and should be considered as approximate values.

### Simulation of the equilibrium protonation of amino acid residues

In order to locate the groups likely to be involved in proton transfer, we have calculated pH titration curves for all the protonatable residues in NDH-2 from *S. aureus* (PDB:4XDB^7^) and from *S. cerevisiae* (PDB:4G73^4^) using methodologies for studying the thermodynamics of proton binding described before in detail^20^,^21^. These methodologies use a combination of Poisson-Boltzmann (PB) calculations, performed with the program MEAD (version 2.2.9)^22^,^23^,^24^, and Metropolis Monte Carlo (MC) simulations, using the program PETIT (version 1.5)^21^. For the *S. aureus* enzyme, the PB/MC calculations were done with the flavin adenine dinucleotide group in two fixed oxidation states: the fully oxidized (FAD) and the fully reduced (FADH_2_) states. For the *S. cerevisiae* enzyme, three systems were simulated: the protein with FAD, the protein with FADH_2_ and the protein with FADH_2_-NAD^+^ charge transfer complex at the catalytic site.

In our calculations, only the crystallographic water molecules with a relative accessibility to the solvent lower than 50% were retained. The relative accessibility of water molecules was computed using the program ASC^25^,^26^. The atomic partial charges and radii used in the PB calculations, for the protein and FAD, FADH_2_ and NAD^+^, were derived from the GROMOS 54A7 force field^27^ using the procedure described in ref. 28. The molecular surface was defined with a solvent probe of 1.4 Å radius and a Stern (ion-exclusion) layer of 2.0 Å. The dielectric constant was 10 for the protein/FAD and 80 for the solvent, the temperature was 300 K and the ionic strength 0.25 M. The finite-difference linear PB calculations used a three-step focusing^29^ procedure employing consecutive grid spacing of 1.0, 0.5 and 0.25 Å.

The MC calculations were done with FAD in fixed oxidation states, and with steps of 0.2 pH units. Each MC simulation comprises 10^5^ MC steps and the acceptance/rejection of each step followed a Metropolis criterion^30^ using the previously determined PB free energies. Each MC step consists of a first cycle of random changes of the protonation states (including tautomeric forms) of all individual sites, followed by a cycle of random double changes of the protonation states of all pairs of sites considered to be strongly coupled; a pair of sites is assumed to be strongly coupled when the electrostatic interaction of at least one of their state combinations is above 2.0 p*K*_a_ units^21^,^31^.

## Additional Information

**How to cite this article**: Marreiros, B. C. *et al*. Structural and Functional insights into the catalytic mechanism of the Type II NADH:quinone oxidoreductase family. *Sci. Rep.* **7**, 42303; doi: 10.1038/srep42303 (2017).

**Publisher's note:** Springer Nature remains neutral with regard to jurisdictional claims in published maps and institutional affiliations.

## Supplementary Material

Supplementary Information
