# Supplementary material for: Structural and Functional insights into the catalytic mechanism of the Type II NADH:quinone oxidoreductase family
Source: Sci Rep. 2017 Feb 9;7:42303. doi: 10.1038/srep42303 (PMC5299459; doi:10.1038/srep42303)
Supplement: Supplementary Information [file srep42303-s1.pdf]

Supplementary information

Structural and Functional insights into the catalytic mechanism of the Type II NADH:quinone oxidoreductase family

Bruno C. Marreiros[1], Filipa V. Sena[1], Filipe M. Sousa[1], A. Sofia F. Oliveira[1], Cláudio M. Soares[1], Ana P. Batista[1,2] and Manuela M. Pereira[1]*

1- Instituto de Tecnologia Química e Biológica – António Xavier, Universidade Nova de Lisboa, Av. da Republica EAN, 2780-157 Oeiras, Portugal

2- Current address: iBET, Instituto de Biologia Experimental e Tecnológica, apartado 12, 2780-901 Oeiras, Portugal

*Corresponding author. Telephone: +351-214469312. Fax: +351-214469314; E-mail: mpereira@itqb.unl.pt.

Supplementary Table 1: Simulated pH titrations of NDH-2 from *S. aureus*. Calculated protonated fractions for the different amino acid residues of NDH-2 from *S. aureus* at different pHs for: A) oxidized state of the protein (FAD); B) reduced state of the protein ($FADH_2$); C) Variation of the protonated fraction between FAD and $FADH_2$ states for each amino acid residue. For the first two panels the blue-red scale represents the protonated fraction from 0 % (blue) to 100 % (red). For panel C) blue represents a negative variation and red a positive variation of the protonated fraction between FAD and $FADH_2$ states. At pH 7, the three residues that appear to suffer the highest variation upon FAD reduction and protonation are E_{172} (25 %), E_{176} (-12 %) and H_{51} (3 %).

A

Proton occupancy (%)

	Amino acid	pH 4	pH 5	pH 6	pH 7	pH 8	pH 9
		FAD	FAD	FAD	FAD	FAD	FAD
YD	D103	14%	2%	0%	0%	0%	0%
GD	D302	93%	76%	40%	12%	3%	0%
2nd Proton conductive pathway	H51	15%	15%	12%	10%	5%	1%
	E52	0%	0%	0%	0%	0%	0%
	E172	78%	65%	45%	32%	16%	4%
	E176	96%	91%	75%	52%	26%	6%
	D179	11%	3%	1%	0%	0%	0%
	E183	43%	11%	2%	0%	0%	0%
1st Proton conductive pathway	K23	100%	100%	100%	99%	96%	81%
	E327	0%	0%	0%	0%	0%	0%
	K331	100%	100%	100%	100%	98%	85%
	K389	100%	100%	99%	95%	73%	30%
	H44	97%	89%	64%	24%	5%	1%
	E46	1%	1%	0%	0%	0%	0%
connection	K379	100%	100%	100%	100%	100%	100%
	D383	0%	0%	0%	0%	0%	0%
others	Y15	100%	100%	100%	100%	100%	100%

B

	Amino acid	pH 4	pH 5	pH 6	pH 7	pH 8	pH 9
		$FADH_2$	$FADH_2$	$FADH_2$	$FADH_2$	$FADH_2$	$FADH_2$
YD	D103	14%	2%	0%	0%	0%	0%
GD	D302	92%	77%	40%	11%	2%	0%
2nd Proton conductive pathway	H51	30%	19%	11%	7%	3%	1%
	E52	1%	0%	0%	0%	0%	0%
	E172	43%	24%	11%	7%	3%	1%
	E176	96%	93%	85%	64%	28%	5%
	D179	12%	3%	1%	0%	0%	0%
	E183	44%	12%	2%	0%	0%	0%
1st Proton conductive pathway	K23	100%	100%	100%	99%	95%	81%
	E327	0%	0%	0%	0%	0%	0%
	K331	100%	100%	100%	100%	97%	86%
	K389	100%	100%	99%	95%	72%	28%
	H44	97%	90%	66%	25%	5%	1%
	E46	1%	1%	0%	0%	0%	0%
connection	K379	100%	100%	100%	100%	100%	100%
	D383	0%	0%	0%	0%	0%	0%
others	Y15	100%	100%	100%	100%	100%	100%

C

	Amino acid	pH 4	pH 5	pH 6	pH 7	pH 8	pH 9
		Variation between FAD and $FADH_2$ states					
YD	D103	0%	0%	0%	0%	0%	0%
GD	D302	1%	-1%	0%	0%	1%	0%
2nd Proton conductive pathway	H51	-15%	-5%	1%	3%	2%	1%
	E52	0%	0%	0%	0%	0%	0%
	E172	35%	41%	34%	25%	14%	3%
	E176	0%	-2%	-9%	-12%	-2%	1%
	D179	-1%	-1%	0%	0%	0%	0%
	E183	-1%	-1%	0%	0%	0%	0%
1st Proton conductive pathway	K23	0%	0%	0%	0%	0%	0%
	E327	0%	0%	0%	0%	0%	0%
	K331	0%	0%	0%	0%	0%	-1%
	K389	0%	0%	0%	0%	1%	2%
	H44	0%	-1%	-2%	-1%	0%	0%
	E46	0%	0%	0%	0%	0%	0%
connection	K379	0%	0%	0%	0%	0%	0%
	D383	0%	0%	0%	0%	0%	0%
others	Y15	0%	0%	0%	0%	0%	0%

Supplementary Table 2: Distances between the amino acid residues composing the proton pathway in the second dinucleotide binding domain present in the three NDH-2s with known structures: A) *S. aureus*; B) *S. cerevisiae*; C) *C. thermarum*. Analyzing the X-ray crystallographic structures available of the three NDH-2s , we have observed the following combinations: in *S. aureus* (PDB:4XDB), E_{183} to D_{179} (6.2 Å, at the surface), D_{179} to backbone of S_{358} (3.3 Å), backbone of S_{358} to E_{176} (4.1 Å), E_{176} to S_{355} (3.5 Å), S_{355} to H_{51} (2.2 Å) and H_{51} to E_{172} (3.7 Å); in *C. thermarum* (PDB:4NWX), E_{180} to D_{176} (9.6 Å, at the surface), D_{176} to E_{173} (5.4 Å), E_{173} to R_{355} (1.8 Å), R_{355} to H_{49} (3.1 Å) and H_{49} to E_{169} (3.8 Å); and in *S. cerevisiae* (PDB: 4G73), D_{254} to R_{507} (2.0 Å, at the surface), R_{507} to D_{249} (3.8 Å) and D_{249} to Y_{449} (2.4 Å). Y_{449} is at 5.2 Å from E_{246} and at 9.4 Å from E_{242}, and may work as the functional replacement for X_{51}, since in NDH-2 from *S. cerevisiae* (and most of eukaryotic NDH-2s) an equivalent to H_{51} is not present to connect the last two glutamate residues (E_{242} and E_{246}). This connection could be hypothesized to be performed under a rotamer change state of Y_{449} (corresponding to S_{355} in *S. aureus*); or conformational changes at S_{96} (corresponding to E_{52} in *S. aureus*) since there are two proline residues before that could unwound α-helix 2 and move S_{96} closer to E_{242}; or by the presence of a structural water molecule, which curiously is observed between $P_{95,}$ E_{242} and E_{246} residues in both NDH-2 structures of *S. cerevisiae* [1-2].

S. aureus		*S. cerevisiae*		*C. thermarum*	
residues	distance (Å)	residues	distance (Å)	residues	distance (Å)
E183 → D179	6.2	D254 → R507	2.0	E180 → D176	9.6
D179 → S358	3.3	R507 → D249	3.8	D176 → E173	5.4
S358 → E176	4.1	D249 → Y449	2.4	E173 → R355	1.8
E176 → S355	3.5	Y449 → E246	5.2	R355 → H49	3.1
S355 → H51	2.2	Y449 → E242	9.4	H49 → E169	3.8
H51 → E172	3.7				

Supplementary Table 3: Simulated pH titrations of NDH-2 from *S. cerevisiae*. Calculated protonated fractions for the different amino acid residues from NDH-2 from *S. cerevisiae* at different pHs for: A) oxidized state of the protein (FAD); B) reduced state of the protein ($FADH_2$); C) Variation of the protonated fraction between FAD and $FADH_2$ states for each amino acid residue; D) oxidized state of the protein (FAD); E) reduced state of the protein with bound NAD^+ ($FADH_2$-NAD^+); F) Variation of the protonated fraction between FAD and $FADH_2$-NAD^+ states for each amino acid residue. For panels A), B), D) and E) blue-red scale represents the protonated fraction from 0 % (blue) to 100 % (red). For panels C) and F) blue represents a negative variation and red a positive variation of the protonated fraction between FAD and $FADH_2$ states and FAD and $FADH_2$-NAD^+ respectively. At pH 5, the three residues that appear to suffer the highest variation upon FAD reduction and protonation (with bound NAD^+) are E_{242} (34 %), E_{246} (-2 %) and D_{383} (-31 %).

A

Proton occupancy (%)

	Amino acid	pH 4	5	6	7	8	9
		FAD	FAD	FAD	FAD	FAD	FAD
YD	Y169	100%	100%	100%	100%	100%	100%
	D170	39%	12%	2%	0%	0%	0%
GD	D383	65%	38%	20%	7%	1%	0%
2nd Proton conductive pathway	E242	55%	35%	12%	3%	0%	0%
	E246	29%	18%	6%	2%	0%	0%
	D249	0%	0%	0%	0%	0%	0%
	D254	10%	2%	0%	0%	0%	0%
	Y449	100%	100%	100%	100%	100%	100%
1st Proton conductive pathway	H71	86%	60%	32%	10%	2%	0%
	D73	0%	0%	0%	0%	0%	0%
	H397	87%	68%	47%	18%	3%	0%
	E401	5%	1%	0%	0%	0%	0%
	K405	100%	100%	99%	96%	84%	68%
	D408	9%	1%	0%	0%	0%	0%
connection	Y482	100%	100%	100%	100%	100%	100%
QBS	E399	91%	78%	57%	22%	3%	0%

B

pH 4	5	6	7	8	9
$FADH_2$	$FADH_2$	$FADH_2$	$FADH_2$	$FADH_2$	$FADH_2$
100%	100%	100%	100%	100%	100%
40%	12%	2%	0%	0%	0%
63%	37%	17%	5%	1%	0%
36%	18%	5%	1%	0%	0%
36%	22%	6%	1%	0%	0%
0%	0%	0%	0%	0%	0%
10%	2%	0%	0%	0%	0%
100%	100%	100%	100%	100%	100%
86%	60%	32%	10%	2%	0%
0%	0%	0%	0%	0%	0%
87%	68%	46%	16%	3%	1%
4%	1%	0%	0%	0%	0%
100%	100%	99%	96%	85%	68%
9%	2%	0%	0%	0%	0%
100%	100%	100%	100%	100%	100%
90%	77%	56%	22%	3%	0%

C

pH 4	5	6	7	8	9
Variation between FAD and $FADH_2$ states					
0%	0%	0%	0%	0%	0%
-1%	0%	0%	0%	0%	0%
2%	1%	3%	2%	0%	0%
19%	17%	7%	1%	0%	0%
-7%	-3%	0%	0%	0%	0%
0%	0%	0%	0%	0%	0%
0%	0%	0%	0%	0%	0%
0%	0%	0%	0%	0%	0%
0%	0%	0%	0%	0%	0%
0%	0%	0%	0%	0%	0%
0%	0%	1%	2%	0%	0%
0%	0%	0%	0%	0%	0%
0%	0%	0%	0%	-1%	0%
0%	0%	0%	0%	0%	0%
0%	0%	0%	0%	0%	0%
0%	1%	1%	1%	0%	0%

D

Proton occupancy (%)

	Amino acid	pH 4	5	6	7	8	9
		FAD	FAD	FAD	FAD	FAD	FAD
YD	Y169	100%	100%	100%	100%	100%	100%
	D170	39%	12%	2%	0%	0%	0%
GD	D383	65%	38%	20%	7%	1%	0%
2nd Proton conductive pathway	E242	55%	35%	12%	3%	0%	0%
	E246	29%	18%	6%	2%	0%	0%
	D249	0%	0%	0%	0%	0%	0%
	D254	10%	2%	0%	0%	0%	0%
	Y449	100%	100%	100%	100%	100%	100%
1st Proton conductive pathway	H71	86%	60%	32%	10%	2%	0%
	D73	0%	0%	0%	0%	0%	0%
	H397	87%	68%	47%	18%	3%	0%
	E401	5%	1%	0%	0%	0%	0%
	K405	100%	100%	99%	96%	84%	68%
	D408	9%	1%	0%	0%	0%	0%
connection	Y482	100%	100%	100%	100%	100%	100%
QBS	E399	91%	78%	57%	22%	3%	0%

E

pH 4	5	6	7	8	9
$FADH_2$-NAD^+	$FADH_2$-NAD^+	$FADH_2$-NAD^+	$FADH_2$-NAD^+	$FADH_2$-NAD^+	$FADH_2$-NAD^+
100%	100%	100%	100%	100%	100%
40%	12%	2%	0%	0%	0%
89%	69%	45%	21%	4%	1%
2%	1%	0%	0%	0%	0%
46%	21%	5%	1%	0%	0%
0%	0%	0%	0%	0%	0%
10%	2%	0%	0%	0%	0%
100%	100%	100%	100%	100%	100%
86%	60%	33%	10%	2%	0%
0%	0%	0%	0%	0%	0%
86%	65%	43%	15%	3%	0%
5%	1%	0%	0%	0%	0%
100%	100%	99%	96%	85%	68%
9%	2%	0%	0%	0%	0%
100%	100%	100%	100%	100%	100%
91%	75%	51%	24%	5%	1%

F

pH 4	5	6	7	8	9
Variation between FAD and $FADH_2$ states					
0%	0%	0%	0%	0%	0%
-1%	0%	0%	0%	0%	0%
-24%	-31%	-25%	-14%	-3%	0%
53%	34%	12%	3%	0%	0%
-17%	-2%	1%	0%	0%	0%
0%	0%	0%	0%	0%	0%
0%	0%	0%	0%	0%	0%
0%	0%	0%	0%	0%	0%
0%	0%	-1%	0%	0%	0%
0%	0%	0%	0%	0%	0%
1%	3%	4%	3%	0%	0%
0%	0%	0%	0%	0%	0%
0%	0%	0%	0%	0%	0%
0%	0%	0%	0%	0%	0%
0%	0%	0%	0%	0%	0%
0%	3%	6%	-2%	-2%	0%

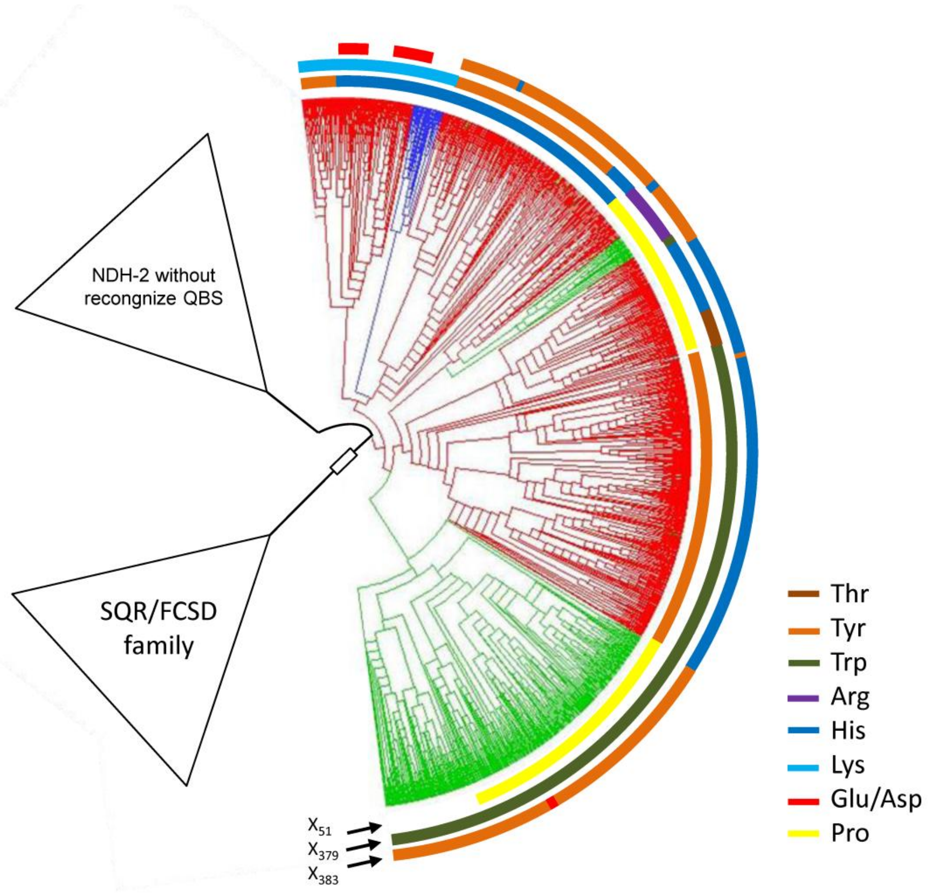

Supplementary Figure 1: Distribution of the three amino acid residues involved in proton transfer from the 2nd dinucleotide binding domain to the quinone. Neighbor-joining dendrogram of NDH-2s family was obtained using 2567 sequences from NDH-2s and sequences from SQRs/FCCDs to define the outer group, midpoint root, 1000 replicates of bootstrap, 999 generated seeds [3]. Branches are colored according to the three domains of life from which the organism containing the respective NDH-2 originates: green corresponds to organisms from Eukarya, red from Bacteria and blue from Archaea. The external ring of the dendrogram, in the region of NDH-2s, indicates the distribution of three amino acid residues proposed to be involved in proton transfer (X_{51}, X_{379} and X_{383}). Color code for amino acid residues is indicated in the figure.

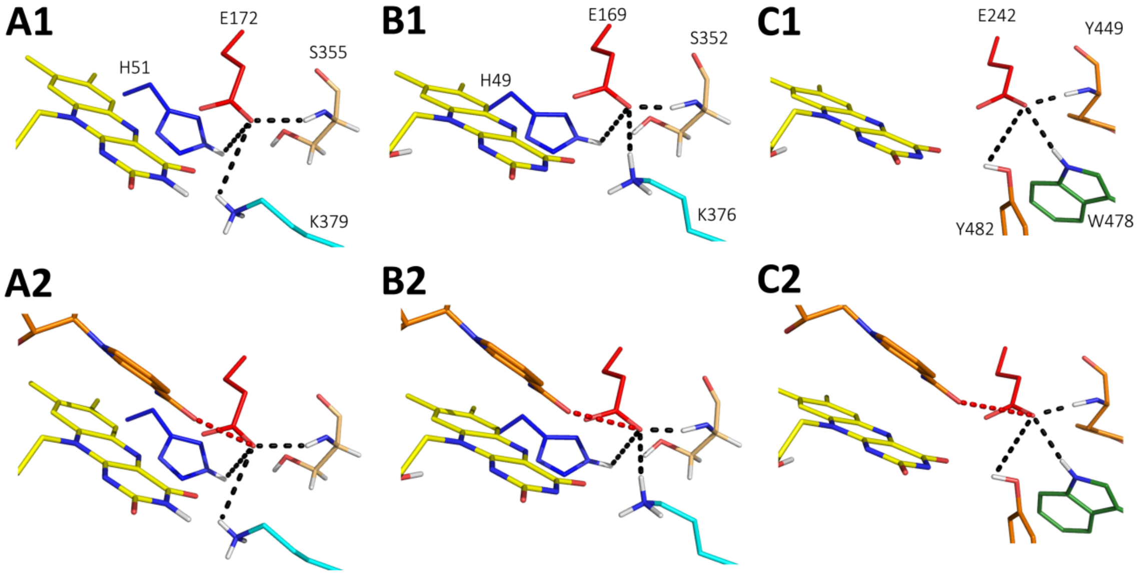

Supplementary Figure 2: Role of glutamate residue (E_{172} in *S. aureus*) in the catalytic mechanism. Cartoon representation of the X-ray crystal structure of NDH-2 from A) *S. aureus* (PDB:4XDB, [4]), B) *C. thermarum* (PDB:4NWZ, [5]) and C) *S. cerevisiae* (PDB:4G73, [2]). Dashed lines show possible hydrogen bonds established by the glutamate residue. A1) E_{172} in *S. aureus* may interact with H_{51}, S_{355} or K_{379} when FAD is oxidized. B1) E_{169} in *C. thermarum* may establish interactions with H_{49}, S_{352} or K_{376} when FAD is oxidized. C1) E_{242} in *S. cerevisiae* may interact with Y_{449}, W_{478} or Y_{482} when FAD is oxidized. A2, B2, and C2) Upon binding of NADH, $FADH_2$-NAD^+ complex is established and possibly a new hydrogen bond with NH_2 from NADH is formed (red dash lines) and others disrupted (black dash line).

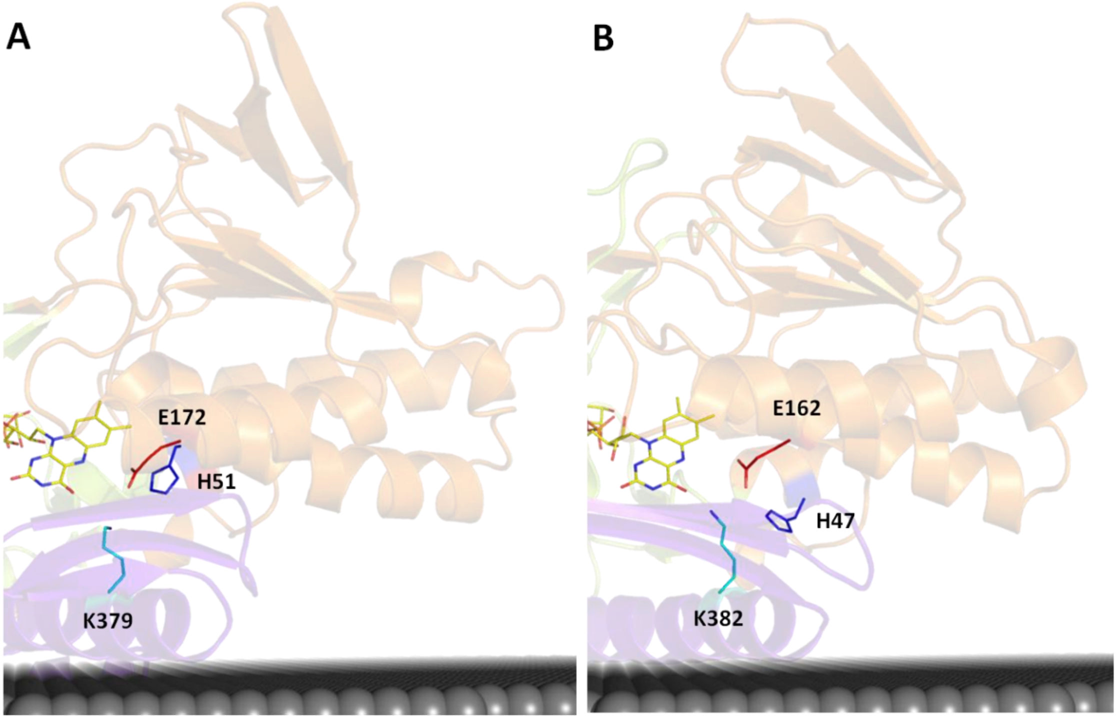

Supplementary Figure 3: Comparison of the 2nd dinucleotide binding domain proton pathway in NDH-2s and SQRs: Cartoon representation of the X-ray crystal structures of A) NDH-2 from *S. aureus* (PDB:4XDB, [4]) and B) SQR from *Aquifex aeolicus* (PDB:3HYW, [6]) (RMSD=3.2 Å). Residues E_{162}, H_{47} and K_{382} occupy the same structural place in SQR as E_{172}, H_{51} and K_{379} in NDH-2. Moreover, K382 at X_{379} equivalent position was suggested to be a proton donor to the quinone [6-7]. We observe that this lysine residue is at least 62 % conserved among SQRs [3].

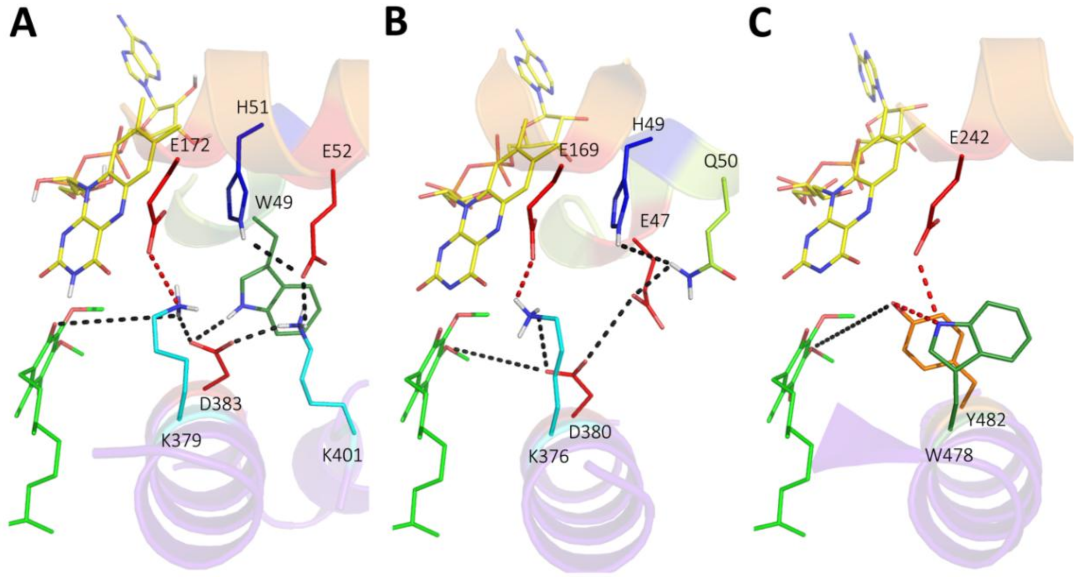

Supplementary Figure 4: Proton conducting elements from NADH binding domain, X_{172} (E_{172}) to the quinone binding pocket. Cartoon representation of the X-ray crystal structure of NDH-2 from A) *S. aureus* (PDB:4XDB, [4]), B) *C. thermarum* (PDB:4NWZ, [5]) and C) *S. cerevisiae* (PDB:4G73, [2]). Red dash lines represent direct proton transfer to X_{379}/ X_{383}. Black dash lines schematize alternative proton transfer to X_{379}/ X_{383} trough X_{51}. A) In NDH-2 from *S. aureus,* E_{172} could release a proton to K_{379} or a proton could be transferred to the quinone binding pocket through H_{51} to E_{52} (~3.3 Å), E_{52} to K_{401} (~2.8 Å) and K_{401} to D_{383} (~2.4 Å). The latter seems to be stabilized by K_{379} (~2.8 Å) and W_{49} (~3.5 Å). B) In the case of NDH-2 from *C. thermarum*, H_{49} (X_{51}) can be linked via Q_{50} (~3.9 Å), Q_{50} to E_{47} (~2.5 Å) and E_{47} to D_{380} (~3.8 Å). D_{380} may be stabilized by K_{376} at ~3.7 Å. C) For NDH-2 from *S. cerevisiae,* a proline is present at X_{51}, and therefore we observed only one possible pathway through a hydrogen bond between E_{242} and $Y_{482.}$

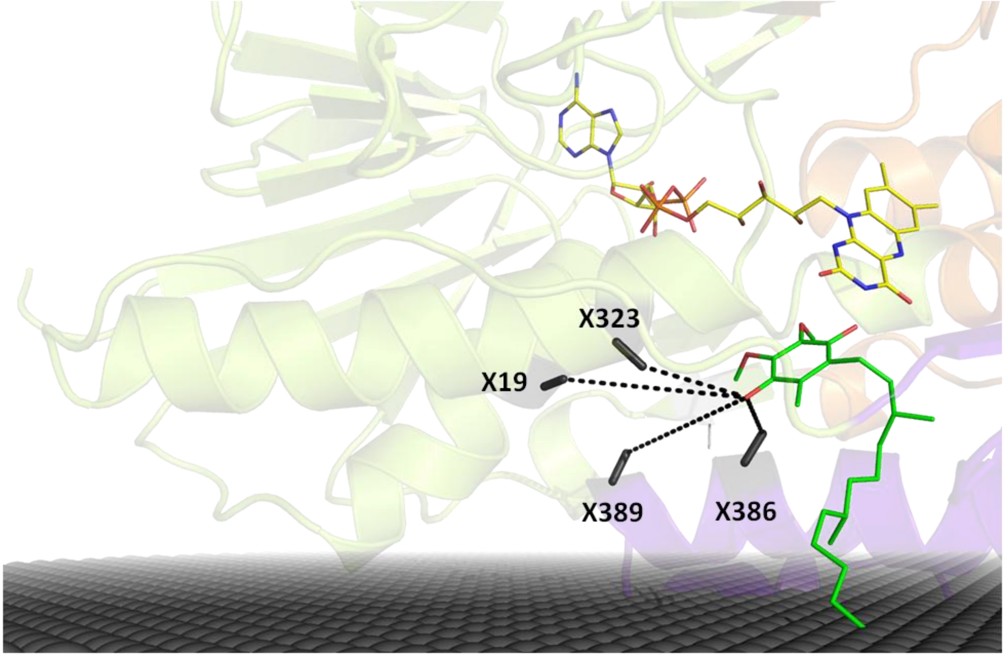

Supplementary Figure 5: Schematic representation of residues which side chains may occupy the same structurally position of H_{397} (X_{323}) side chain. The positions are: X_{19} (*S. aureus*), located after the first conserved $G_{12}xGxxG_{17}$ motif and which can be occupied by a lysine (3 %), an arginine (9 %), a tyrosine (14 %) or a glutamate (18 %); X_{323} (*S. aureus*) which is a lysine (6 %), glutamate (8 %) or an histidine (17 %); X_{386} or X_{389}, which are located at the end of the first amphipathic α-helix (Figure 1C) and may be occupied by a lysine residue (X_{389} corresponding to K_{389} in *S. aureus*, Figure 7A1) and a tyrosine (X_{386}, corresponding to Y_{383} in *C. thermarum*) (Figure 7B). We observed that 7 % of the NDH-2s have a lysine, an arginine, a histidine or a tyrosine residue at X_{389}, but 58 % NDH-2s have these residues at X_{386}.

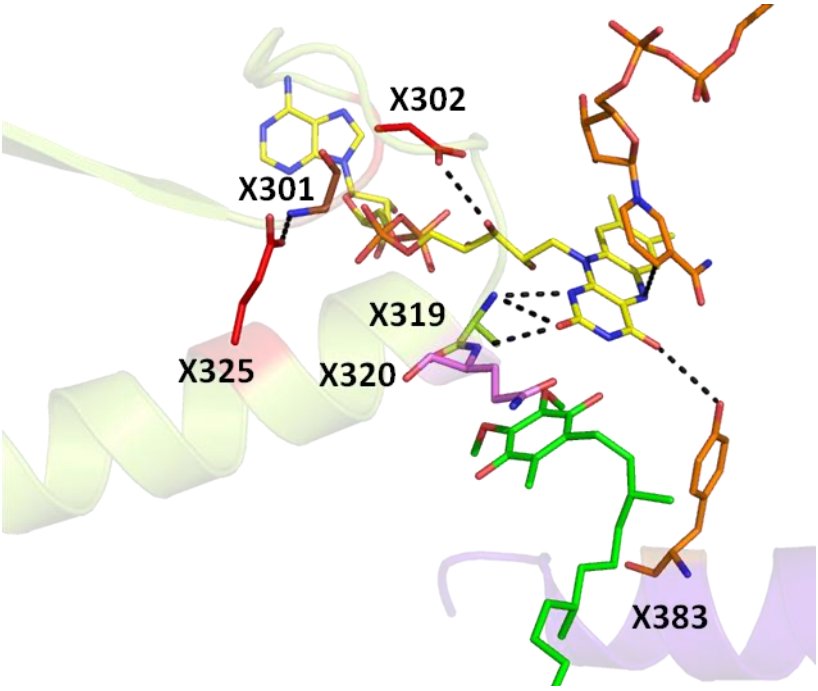

Supplementary Figure 6: Schematic representation of the catalytic site indicating possible proton transfer in the presence of substrates. Some of the residues involved in the stabilization of FAD (yellow), where dashed lines represent hydrogen bonds: backbone of X_{301} (Glycine) interacts with X_{325} (E/Q/H in *S. cerevisiae*, *S. aureus*, *C. thermarum*, respectively); X_{302} (Aspartate) interacts with O3* from FAD; backbones of X_{319} and X_{320} interact with N1 and O2 from FAD isoalloxazine ring; X_{379}/X_{383} (X_{379} is a K in *S. aureus* and *C. thermarum* and X_{383} is a Y in *S. cerevisiae*) interact with O4 from FAD isoalloxazine ring. Schemes based on the NDH-2 co-crystalized with NADH and quinone from *S. cerevisiae* (PDB:4G73 [2]). X_{325} may be considered as a critical residue in the propagation of the conformational changes from the GD motif to the QBS motif (caused by the FAD reduction, hence its bending), possibly necessary for the quinone protonation.

References

1 Iwata, M. *et al.* The structure of the yeast NADH dehydrogenase (Ndi1) reveals overlapping binding sites for water- and lipid-soluble substrates. *Proceedings of the National Academy of Sciences of the United States of America* **109**, 15247-15252, doi:10.1073/pnas.1210059109 (2012).

2 Feng, Y. *et al.* Structural insight into the type-II mitochondrial NADH dehydrogenases. *Nature* **491**, 478-482, doi:10.1038/nature11541 (2012).

3 Marreiros, B. C., Sena, F. V., Sousa, F. M., Batista, A. P. & Pereira, M. M. Type II NADH:Quinone oxidoreductase family: Phylogenetic distribution, Structural diversity and Evolutionary divergences. *Environmental microbiology*, doi:10.1111/1462-2920.13352 (2016).

4 Sena, F. V. *et al.* Type-II NADH:quinone oxidoreductase from Staphylococcus aureus has two distinct binding sites and is rate limited by quinone reduction. *Molecular microbiology* **98**, 272-288, doi:10.1111/mmi.13120 (2015).

5 Heikal, A. *et al.* Structure of the bacterial type II NADH dehydrogenase: a monotopic membrane protein with an essential role in energy generation. *Molecular microbiology* **91**, 950-964, doi:10.1111/mmi.12507 (2014).

6 Marcia, M., Ermler, U., Peng, G. & Michel, H. The structure of Aquifex aeolicus sulfide:quinone oxidoreductase, a basis to understand sulfide detoxification and respiration. *Proceedings of the National Academy of Sciences of the United States of America* **106**, 9625-9630, doi:10.1073/pnas.0904165106 (2009).

7 Cherney, M. M., Zhang, Y., Solomonson, M., Weiner, J. H. & James, M. N. Crystal structure of sulfide:quinone oxidoreductase from Acidithiobacillus ferrooxidans: insights into sulfidotrophic respiration and detoxification. *Journal of molecular biology* **398**, 292-305, doi:10.1016/j.jmb.2010.03.018 (2010).
